# Screening of Crucial Cytosolicproteins Interconnecting the Endoplasmic Reticulum and Mitochondria in Parkinson’s Disease and the Impact of Anti-Parkinson Drugs in the Preservation of Organelle Connectivity

**DOI:** 10.3390/brainsci13111551

**Published:** 2023-11-05

**Authors:** Athira Anirudhan, S. Mahema, Sheikh F. Ahmad, Talha Bin Emran, Shiek S. S. J. Ahmed, Prabu Paramasivam

**Affiliations:** 1 Central Research Laboratory, Believers Church Medical College Hospital, Kuttapuzha, Thiruvalla 689101, Kerala, India; 2 Drug Discovery and Multi-Omics Laboratory, Faculty of Allied Health Sciences, Chettinad Academy of Research and Education, Chettinad Hospital and Research Institute, Kelambakkam 603103, Tamil Nadu, India; 3 Department of Pharmacology and Toxicology, College of Pharmacy, King Saud University, Riyadh 11451, Saudi Arabia; 4 Department of Pathology and Laboratory Medicine, Warren Alpert Medical School, Brown University, Providence, RI 02912, USA; 5 Legorreta Cancer Center, Brown University, Providence, RI 02912, USA; 6 Department of Pharmacy, Faculty of Allied Health Sciences, Daffodil International University, Dhaka 1207, Bangladesh; 7 Madras Diabetes Research Foundation and Dr. Mohan’s Diabetes Specialities Centre, WHO Collaborating Centre for Non-Communicable Diseases Prevention and Control & IDF Centre of Education, Gopalapuram, Chennai 602105, Tamil Nadu, India; 8 Department of Neurology, School of Medicine, University of New Mexico Health Sciences Center, Albuquerque, NM 87131, USA

**Keywords:** Parkinson’s disease, mitochondrial-ER, mitochondria-associated membranes (MAMs), systems biology, neuroinflammation

## Abstract

Mitochondrial dysfunction is well-established in Parkinson’s disease (PD); however, its dysfunctions associating with cell organelle connectivity remain unknown. We aimed to establish the crucial cytosolic protein involved in organelle connectivity between mitochondria and the endopalmic reticulum (ER) through a computational approach by constructing an organelle protein network to extract functional clusters presenting the crucial PD protein connecting organelles. Then, we assessed the influence of anti-parkinsonism drugs (*n* = 35) on the crucial protein through molecular docking and molecular dynamic simulation and further validated its gene expression in PD participants under, istradefylline (*n* = 25) and amantadine (*n* = 25) treatment. Based on our investigation, D-aspartate oxidase (DDO )protein was found to be the critical that connects both mitochondria and the ER. Further, molecular docking showed that istradefylline has a high affinity (−9.073 kcal/mol) against DDO protein, which may disrupt mitochondrial-ER connectivity. While amantadine (−4.53 kcal/mol) shows negligible effects against DDO that contribute to conformational changes in drug binding, Successively, DDO gene expression was downregulated in istradefylline-treated PD participants, which elucidated the likelihood of an istradefylline off-target mechanism. Overall, our findings illuminate the off-target effects of anti-parkinsonism medications on DDO protein, enabling the recommendation of off-target-free PD treatments.

## 1. Introduction

Parkinson’s disease (PD) is one of the most common movement disorders, characterized by the loss of dopamine-producing neurons in the midbrain’s Substantia nigra region of the human brain [1]. Tremor, bradykinesia, rigidity, and postural instability are the cardinal symptoms that arise gradually with neuron loss that allow PD diagnosis on neurological examination [2]. Neuronal death occurs with the accumulation of misfolded synuclein protein, known as Lewy body, a prominent feature that is directly related to the severity and progression of PD [3]. Notably, synaptic and axonal neuronal degeneration are early pathological events underlying symptoms during the onset of PD [4]. To date, several PD risk factors have been reported. Genetic mutations and environmental toxins are the most contributing factors that disrupt mitochondrial function, causing oxidative damage to neuronal cells. Furthermore, calcium homeostasis, cellular proteostasis, axonal transport, and mitochondrial-endoplasmic trafficking have been suggested to play a role in PD pathogenesis [5].

In eukaryotic cells, the compartmentalization of organelles and their interdependencies play a crucial role in the regulation of numerous metabolic and physiological processes. Such regulation between the organelles is influenced by external and internal responses carried out by membrane receptors, signaling proteins, metabolites, and ions [6]. ER-mitochondria interactions are essential for optimal physiological cell activity. Mitochondria communicate bidirectionally with endoplasmic reticulum, peroxisomes, and lysosomes via mitochondrial contact sites in order to maintain cellular homeostasis, redox activity, calcium homeostasis, iron-sulfur biogenesis, amino acid metabolism, and fatty acid oxidation [7]. Meanwhile, miscommunication between the ER and mitochondria results in cellular dyshomeostasis. Also, the mitochondrial dysfunction leads to endolysososmal defects as well as ER stress [8]. In order to maintain proper neural functioning, mitochondria are essential for producing the majority of cellular ATP. These communications between organelles and their coordination are essential for the survival of all cells, including neurons [9]. However, the research involving organelle communication and the regulatory mechanism underlying neuronal death in PD remains to be elucidated.

Multiple sources indicate that mitochondrial dysfunction plays a crucial role in neurodegenerative processes in Parkinson’s [10] and Alzheimer’s disease (AD) [11]. Mitochondrial dysfunction can affect the communication organelles of a cell. Notably, the mitochondria and endoplasmic reticulum (ER) are interconnected by mitochondria-associated membranes (MAMs), which regulate multiple cellular physiological processes [12]. MAMs play a role in the transport of signaling proteins and metabolites for organelle function in neurons [13,14]. According to Gómez-Suaga et al. (2018), the loss of ER-mitochondria communication affects normal cellular physiology, which promotes PD [15]. Also, reports indicate that environmental toxins such as rotenone and manganese alter the function of MAMs, resulting in PD [16,17,18,19]. Similarly, animal studies on PD indicate that the loss of connectivity between ER and mitochondria promotes the degeneration of dopamine-producing neurons [20,21]. Loss of mitochondrial-ER connectivity generates reactive oxygen species (ROS) and promotes oxidative cell damage [22]. On the basis of these studies, we aimed to investigate mitochondrial-ER connectivity by means of cytosolic protein signals to determine their role in the pathogenesis of Parkinson’s disease. Simultaneously, we attempted to identify potential PD drugs that could maintain organelle connection without inhibiting the essential mitochondrial-ER connecting protein.

**Figure 1:**
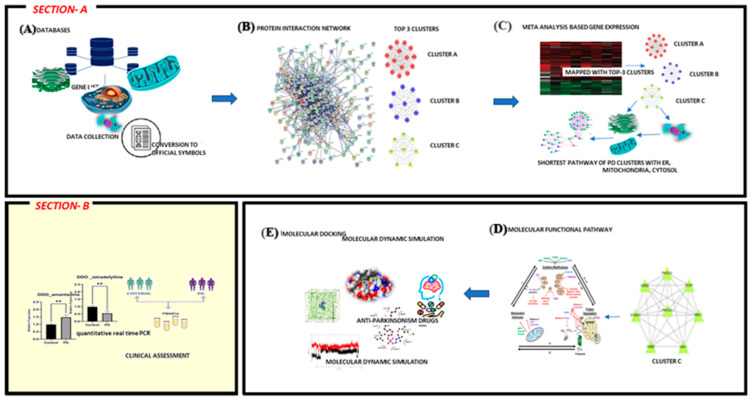
Comprehensive overview of the current investigation: Section-A: collection of organelle-specific proteins of the endoplasmic reticulum (ER), mitochondria, and cytosol from various databases and conversion of the collected proteins into official symbols in order to build a protein interaction network. The top three clusters were extracted from the protein network using MCODE clustering. Simultaneously, meta-gene expression analysis was carried out with PD datasets retrieved from the NCBI-GEO datasets. The differentially expressed genes from the meta-analysis were mapped to the top three clusters to select clusters influenced by PD pathogenesis (termed PD cluster) and screened to select the cluster presenting proteins localized to the mitochondria, endoplasmic reticulum, and cytosol. Then, protein shortest path analysis was conducted to discover the crucial cytosolic protein connecting the mitochondria and endoplasmic reticulum (ER). Further, molecular docking and molecular dynamic (MD) simulation were performed to evaluate the impact of anti-Parkinson’s disease drugs on the crucial cytosolic protein. Section-B: To validate our finding, qPCR was used to measure gene expression in peripheral blood mononuclear cells (PBMC) of PD drug-treated groups and controls for the selected gene, and the significance was assessed by statistical analysis.

Herein, we implement a series of computational approaches to establish the protein signal that mediates cross-talk between mitochondria and the ER (Figure 1). Through computation, we found that the D-aspartate oxidase (DDO) protein is a key protein in the connection between mitochondria and the endoplasmic reticulum (ER). Using molecular docking and molecular dynamic (MD) simulation, we also found out how 35 anti-parkinsonism drugs affected the DDO protein. Our results suggest that istradefylline may block the DDO, which may inhibit mitochondrial-ER connectivity. In contrast, amantadine had minimal effects on DDO, which could be advantageous for the treatment of PD. Further, the DDO gene expression in PD participants under istradefylline or amantadine treatment was assessed, which established the likelihood that istradefylline has an off-target mechanism.

## 2. Materials and Methods

### 2.1. Data Collection and Protein Interactome Construction

Organelle-specific proteins (endoplasmic reticulum, mitochondria, and cytosol) were collected from the human protein atlas (www.proteinatlas.org) (accessed on 12 August 2023), gene ontology (http://geneontology.org/) (accessed on 17 August 2023) and organelle (http://labs.mcdb.lsa.umich.edu/organelledb/) accessed on 23 August 2023, databases. All the collected proteins were converted into official symbols using the HGNC (https://www.genenames.org/) (accessed on 1 September 2023) database and further verified using BioGPS (http://biogps.org/) (accessed on 7 September 2023) to confirm their organelle specificity based on cellular localization. Then, the protein list was subjected to a protein interaction network using the String plug-in in Cytoscape software 3.8.1 verison with zero external interactions. Using the MCODE clustering algorithm, the crucial clusters were extracted from the constructed protein network.

### 2.2. Cluster Analysis and Pathway Enrichment

Simultaneously, a gene expression-based meta-analysis was performed [23] to determine the differentially expressed genes (DEGs) in the PD brain. Three datasets, GSE28894 (114 samples), GSE8397-GPL97 (94 samples), and GSE20186-GPL96 (69 samples), were selected based on the inclusion and exclusion criteria for the meta-analysis. The inclusion criteria include (a) the dataset presenting a case-control study with Homo sapiens as a study subject, (b) the dataset restricted to brain tissue that compares PD and control, and (c) the dataset containing raw intensity values for meta-analysis. Alternatively, the exclusion criteria for the dataset include (a) animal models and in-vitro studies; (b) datasets without replicate samples in a group; and c) studies other than microarray experiments. The differential gene expression analysis was performed using a limma algorithm-based linear model on microarray data [24], and then Fisher’s test was implemented to calculate the *p*-value. All the collected DEGs were then mapped to the selected clusters to confirm their significance in PD. Such selected clusters with PD genes were termed “PD clusters”. Among the PD clusters, the cluster presenting proteins that are localized at the endoplasmic reticulum, mitochondria, and cytosol was selected and subjected to molecular functional enrichment analysis using the Shinygo database [25]. In addition, the shortest path for cytosol proteins connecting the mitochondria and ER was identified for the chosen PD cluster. Such assessment delivers a crucial cytosol protein that channels mitochondria and the ER.

### 2.3. Molecular Modelling of Crucial Cytosolic Proteins

Next, the three-dimensional structure of the crucial cytosol protein was retrieved from the AlphaFoldDB (Q99489 (OXDD_HUMAN)) due to lack of complete protein structure in Protein Data Bank. The retrieved model structure was optimized using the Protein Prep Wizard of Schrödinger-Maestro 11.2 version. The forcefield energy minimization was implemented by setting the heavy atom RMSD (root mean square deviation) to 0.30 Å with OPLS_2005 [26]. Further, the protein structure was processed to identify the putative ligand binding sites within the protein structure using SiteMap2.6 in Schrodinger Suite. Then a grid around the binding sites was generated using the Receptor Grid Generation module in Schrodinger Suite for molecular docking.

### 2.4. Ligand Preparation and Molecular Docking

Based on the literature survey, a list of anti-parkinsonism drugs was identified, and their structures were downloaded from the Drug Bank (www.drugbank.com) (accessed on 15 September 2023) database (Table 1). The drug structures were optimized using the LigPrep module of Maestro v11.2 in the Schrodinger suite. After the ligand optimization, molecular docking was performed in the standard precision (SP) mode of the GLIDE module of the Maestro v11.2 Schrodinger Suite [27]. The Glide module helps to assess the active interaction between cytosol protein and the PD drug. Based on the docking score, the PD drug presenting both the highest and lowest affinity with the cytosol protein was selected for molecular-dynamic simulation.

### 2.5. Molecular Dynamic Simulation

Three independent MD simulations (1. ligand_free_protein; 2. Ligand (high affinity) with protein 3. Ligand (low affinity) with protein) were performed using the GROMACS 2020.4 version to assess the stability of the protein-drug complex. In GROMOS 54a7, single-point charge (SPC) was implemented to generate topology files for proteins, whereas the drug topology was generated using the PRODRG server. Following the protocol of Robertson et al., 2019, the structure was stimulated by implementing an OPLS-AA/L all-atom forcefield, placing it into the 1.0 nm cubic box, and solvating using TIP3P water [28]. Later, with the addition of sodium or chloride ions, the solvated system was neutralized with a constant salt concentration (0.15 mol/L). Energy minimization steps of the steepest descent method (*n* = 50,000) were adopted, which helped overcome the unfavorable contacts and clashes in the stimulated complex. Similarly, the NVT and NPT equilibrations were performed for energy minimization. Further, the MD run was performed for 100 ns, and the outcome trajectories such as root-mean-square deviation (RMSD), root-mean-square fluctuation (RMSF), radius of gyration (Rg), solvent accessible surface area (SASA), and total number of H-bonds were assessed for the conformational stability of the complexes [29]. Additionally, two parallel runs were performed to confirm the reliability of the simulation.

### 2.6. Patient Recruitment and Clinical Assessment

For validation, fifty participants with Parkinson’s disease under the Group 1: istradefylline (*n* =25) and Group 2: amantadine (*n* =25) treatments were recruited and compared to 25 healthy controls (Group 3) following the inclusion and exclusion criteria. Institutional ethical approval was obtained from the Chettinad Academy of Research and Education, Tamil Nadu, India. Characteristics such as gender, age, and BMI were collected from all the participants. Especially for PD participants with the support of movement disorder specialists, clinical characteristics such as age of disease onset, the Unified Parkinson’s Disease Rating Scale (UPDRS), and the Hoehn & Yahr scale with the presence of any two or more cardinal symptoms were recorded. The exclusion parameters include (a) individuals who show no evidence of dopaminergic neuronal loss and (b) suspected participants who have a secondary cause of Parkinsonism, like the use of neuroleptic agents. Additionally, healthy individuals (free of neurological or neuropsychiatric diseases) were categorized based on a complete neurological examination (Table 1).

### 2.7. Gene Expression Profiling

Based on the treatment regime and clinical characteristics, the enrolled participants were grouped as follows: Group 1: PD under istradefylline + L-DOPA (*n* = 25); Group 2: PD under amantadine + L-DOPA (*n* = 25); and Group 3: Healthy Controls (*n* = 25). From the participants, 3 mL of peripheral blood was collected, and peripheral blood mononuclear cells (PBMC) were isolated using Histopaque-1077 (Sigma-Aldrich, Burlington, MA, USA) reagent. The collected PBMCs for each individual were subjected to RNA isolation using the TRIzol (Invitrogen) reagent, and cDNA was constructed with 100 ng of RNA measured using Nanodrop 2000 (Thermo Scientific, Waltham, MA, USA). The cDNA was constructed with High-Capacity cDNA reverse transcription kit from Thermo Fisher Scientific, Waltham, MA, USA, following the manufacturer’s protocol. Later, using gene-specific primers, quantitative real-time PCR (ABI-7000, Applied Biosystems, USA) was performed for all three groups with GAPDH as the housekeeping gene (Table 2). The gene expressions among the groups were calculated using the delta-delta Ct value method.

### 2.8. Statistical Analysis

The clinical and demographic characteristics of the cohort were compared using the relevant SPSS version 21 statistical methods [30]. The distribution of the collected numerical variables was then analyzed. Because each variable had a normal distribution, parametric analyses were utilized. Age (demographic) and gene expression data were analyzed using a one-way ANOVA followed by Tukey post hoc comparisons for multiple group comparisons (Group-1: 25, Group-2: 25, and Group-3: 25). All values are presented as the mean and standard deviation, and p-values less than 0.05 are regarded as statistical significance.

## 3. Results

### 3.1. Data Collection and Meta-Analysis of Cluster Proteins

Two authors manually collected the list of organelle-specific proteins from various databases. A total of 2941 proteins, 243 (ER), 1399 (mitochondria), and 1299 (cytosol) proteins were retrieved from various sources. The protein list was then verified using BioGPS to validate their cell organelles’ specificity. The whole list of proteins was combined to build a protein interaction network that contains 2918 nodes with 23,210 edges using the String plug-in in Cytoscape software. Further, the network was subjected to the MCODE algorithm to extract the top three highly interconnected functional clusters. Cluster-A had 84 proteins and 3469 edges; cluster-B had 66 proteins and 1844 edges; and cluster-C contained 44 proteins and 450 edges.

### 3.2. Interactome and Pathway Enrichment Analysis

**Figure 2:**
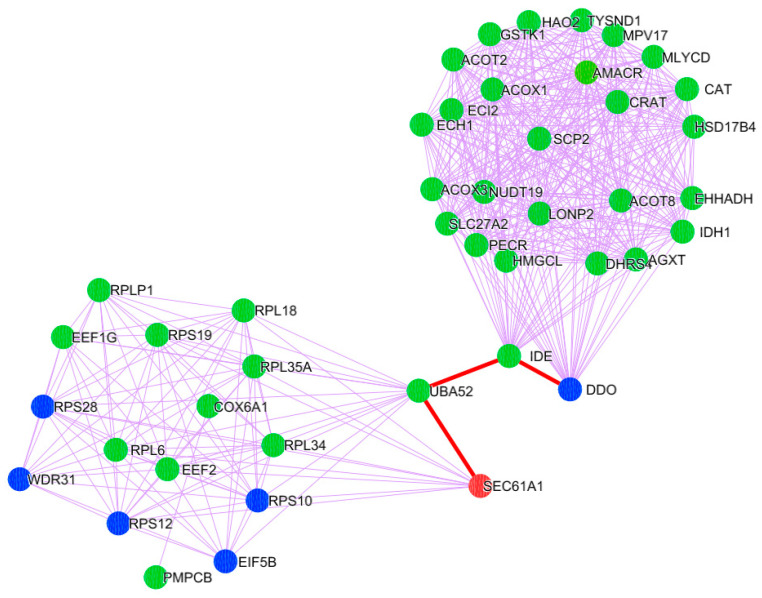
PD Cluster with mitochondrial, ER, and cytosolic proteins. A node represented in orange is the endoplasmic reticulum; a green-colored node represents mitochondrial proteins; and a blue-colored node corresponds to the proteins localized in the cytosol. The red-colored highlighted edges show the inter-cell organelle interaction via protein signal.

**Figure 3:**
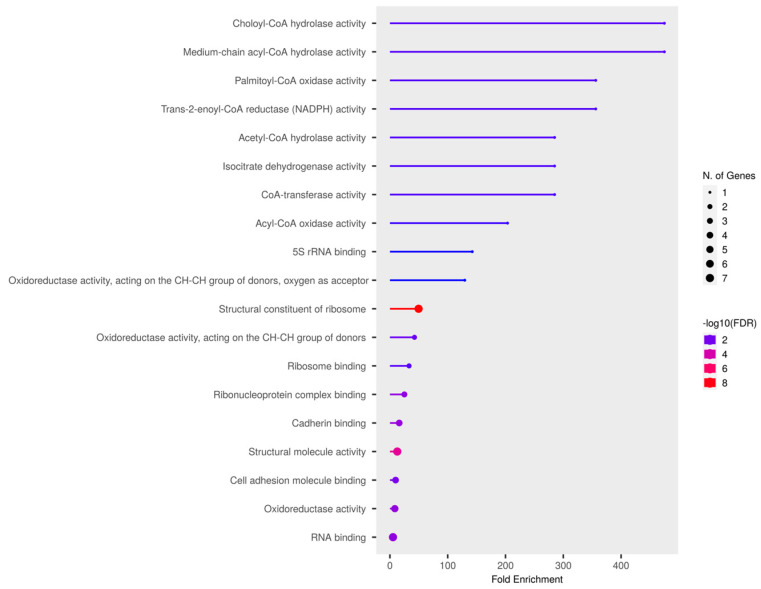
Molecular enrichment analysis of selected PD cluster.

To determine the significance of clusters in PD pathogenesis, the gene expression-based meta-analysis was performed by integrating the datasets GSE28894, GSE8397, and GSE20186. A total of 277 samples were subjected to a meta-analysis in which 3973 genes were differentially expressed (DEGs) in PD compared to control (*p* < 0.05, Fisher’s test). All three clusters showed the presence of PD genes. Further, the clusters presenting mitochondria, ER, and cytosolic proteins were selected. Notably, cluster-C (Figure 2) with 44 proteins showed the presence of 37 mitochondrial, six cytosolic, and one ER protein. Further, the molecular functional enrichment analysis of these 44 proteins reveals their involvement in PD-associated molecular mechanisms (Figure 3) Then, the organelle connectivity within cluster-C was assessed based on the shortest path method to trace protein interactions between mitochondria, endoplasmic reticulum, and cytosol. Our shortest path assessment revealed that DDO (D-aspartate oxidase) a crucial cytosolic protein, interacts with SEC61A1 endoplasmic reticulum protein through the IDE (Insulin Degrading Enzyme) and UBA52 (Ubiquitin A-52 Residue Ribosomal Protein Fusion Product 1) of mitochondrial proteins. Thereby, DDO could be one of the important proteins that channels the mitochondria and endoplasmic reticulum.

### 3.3. Molecular Docking and Molecular Dynamic Simulation

**Figure 4:**
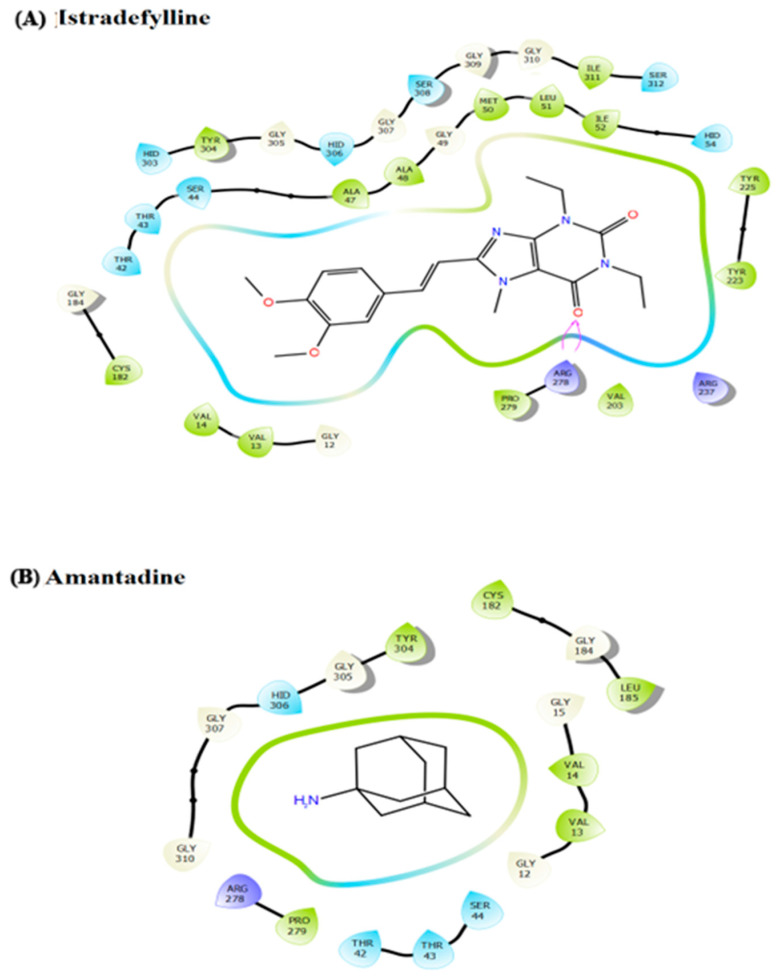
Ligand interaction diagram for the docked anti-Parkinsonism drugs (**A**) istradefylline drug contributes high affinity with DDO cytosol protein, forming a significant number of hydrogen bonds; and (**B**) amantadine drug contributes less affinity with no hydrogen bond formation with the DDO protein.

**Figure 5:**
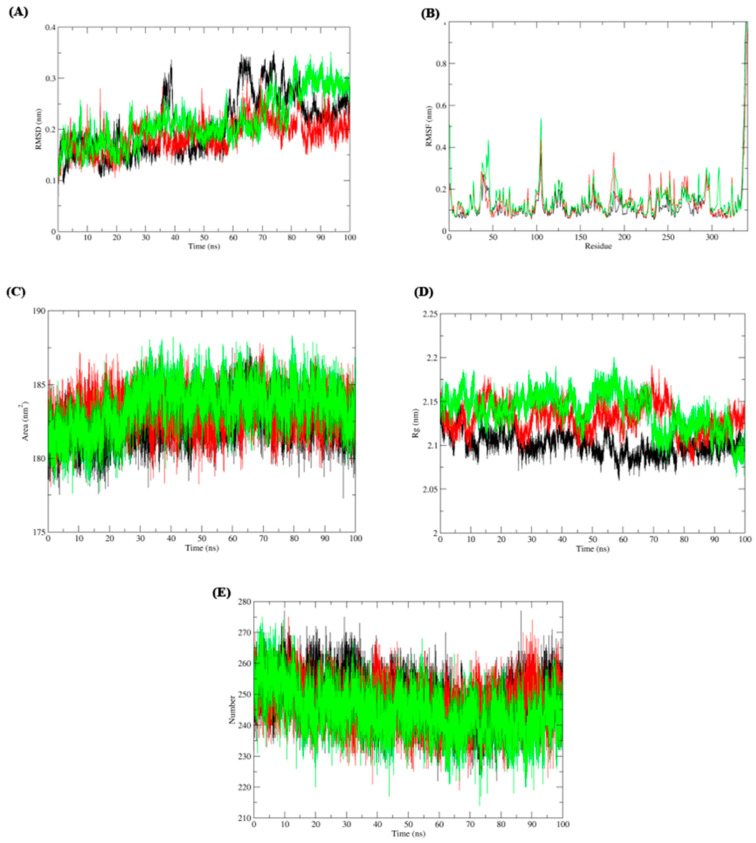
Molecular dynamic simulation of ligand free DDO (colored: black), DDO_istradefylline (colored: red), and (3) DDO_amantadine (colored: green) complexes. (**A**) root-mean-square deviation (RMSD), (**B**) root-mean-square fluctuation (RMSF), (**C**) solvent accessible surface area (SASA), (**D**) radius of gyration (Rg), and (**E**) H-bonds.

Next, the impact of anti-parkinsonism drugs on DDO protein was determined through docking analysis. Out of 35 drugs, istradefylline was noticed to have the highest affinity (−9.073 kcal/mol) with DDO protein, whereas minimal affinity was observed for amantadine with a binding energy of −4.543 kcal/mol (Table 3) (Figure 4). Based on the docking score, MD simulations were run on the DDO_istradefylline and DDO_amantadine complexes and compared with the ligand-free DDO protein to figure out the stability of the protein-drug complexes. The average RMSD (Figure 5A) value of ligand_free_DDO was 0.208 nm, DDO_istradefylline was 0.188 nm, and DDO_amantadine was 0.215 nm. Similarly, the average RMSF (Figure 5B) values were 0.128, 0.141, and 0.135 nm for ligand_free_DDO, DDO_istradefylline, and DDO_amantadine, respectively. Also, the SASA (Figure 5C) average corresponding to ligand_free_DDO was 182.46 nm^2^, DDO_istradefylline was 183.129 nm^2^, and DDO_amantadine was 183.451 nm^2^. Likewise, average Rg (Figure 5D) and HB (Figure 5E) count for ligand_free_DDO (2.10 Å; 249), DDO_istradefylline (2.13 Å; 247), and DDO_amantadine (2.13 Å; 244) (Figure 5).

### 3.4. Gene Expression of DDO Gene in PD

**Figure 6:**
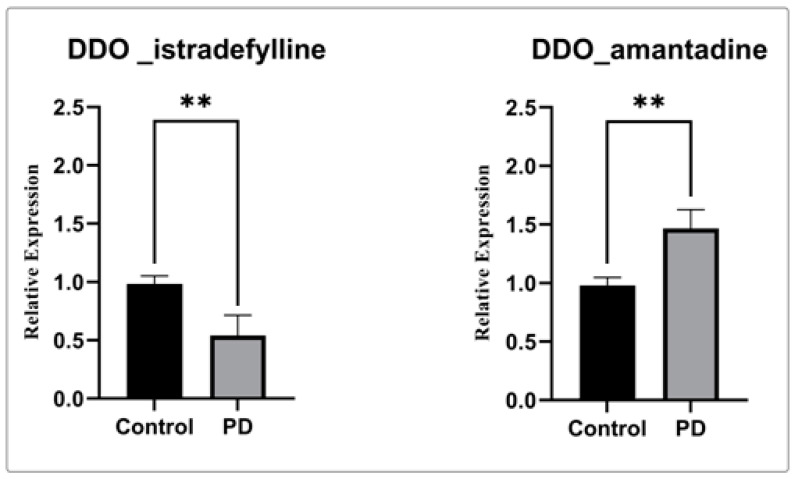
Gene expression analysis of DDO gene with istradefylline and amantadine.The relative expression of DDO shows significantly decreased DDO gene expression when treated with istradefylline. (**) significant changes were observed in the PD participant treated with istradefylline and amantadine compared to the control.

In order to confirm our computational finding, gene expression analysis using real-time PCR was performed for the selected genes in the PBMC of the PD drug-treated groups compared to the control. The gene expression analysis shows significant downregulation of DDO in the treatment of istradefylline when compared to amantadine and control (Figure 6). Henceforth, our data clearly shows usage of the istradefylline anti-parkinsonism drug has a significant impact on mitochondrial and ER connectivity. Whereas, the amantadine showed a minimal effect on DDO expression.

## 4. Discussion

The molecular pathogenesis of PD is complex and is mostly correlated with mitochondrial dysfunction that is linked with the generation of oxidative stress, causing neurodegeneration. However, the mitochondria are not only a single entity; they collaborate with other organelles in a cell to perform normal physiological processes. Notably, the mitochondria and endoplasmic reticulum (ER) are highly connected and exhibit significant associations in the pathological process of PD. Both cellular organelles primarily receive external stimuli through protein signals from the cytoplasm, which cause the organelles to alter their functions. The role of endoplasmic reticulum (ER) and mitochondrial dysfunction in Parkinson’s disease (PD) is defined through several pieces of literature [20]. However, there was still a lack of substantial knowledge about the role of organelle connectivity in the etiology of PD. The maintenance of a robust population of mitochondria and endoplasmic reticulum (ER) is thought to be crucial for supporting neuronal function. However, any disruption to this delicate balance can lead to cellular oxidative stress and ultimately contribute to cellular demise. Therefore, an investigation of the intricate defective molecular process and the unintended effects (off-target) of anti-parkinsonism medications on advantageous proteins may enable us to propose improved pharmaceutical options for the treatment of Parkinson’s disease.

In this study, a series of computational methods were employed to ascertain the cytoplasmic linker that mediates interactions between mitochondria and the endoplasmic reticulum. The methodology employed in our study encompasses the collection of protein-encoding genes that are localized in the mitochondria, cytosol, and endoplasmic reticulum. Then the construction of the interaction network and the identification of the functionally relevant Parkinson’s disease clusters through meta-analysis and pathway enrichment analysis. Subsequent analysis of organelle interconnection using the shortest path method and the utilization of molecular docking and simulation techniques facilitate the comprehension of the stability of the protein-drug complex. Based on our analysis of the interaction network, D-aspartate oxidase (DDO), a cytosolic protein, was mostly interconnected with mictochondria and indirectly associated with ER protein (SEC61A1) [31]. The D-aspartate oxidase (DDO) enzyme is of significant importance in the metabolism of D-aspartate (D-Asp) within the mammalian brain [32,33]. The levels of D-Asp typically exhibit higher concentrations throughout the embryonic and perinatal stages but experience a significant decline during maturity [34]. D-aspartate (D-Asp) participates in glutamatergic neurotransmission by acting as an agonist to glutamate [35]. The research findings indicate that the suppression of the DDO gene results in a heightened level of D-Asp in the brain [35]. Moreover, an elevated concentration of D-Asp in the brain has been linked to an augmentation of NMDAR-dependent long-term potentiation (LTP) in the hippocampus and a reduction in long-term depression (LTD) in the striatum [36,37,38,39]. Excitement of NMDARs promotes synaptic strength and connectivity, the frequent stimulation of the receptor leads to neuronal death [39]. Errico F et al., 2011 show the association of NMDAR receptor overstimulation due to increased D-Asp level causes early breakdown of basal glutamatergic transmission, synaptic plasticity, and hippocampal reference memory in 13/14-month-old Ddo−/− knock downed mice with neuroinflammation and cell death in midbrain dopaminergic neurons, as well as a precocious onset of L-DOPA-induced dyskinesia [39]. Similarly, the loss of excitatory glutamatergic synapses and the reduction of synaptic GluN1 and GluN2B subunits were noticed [40]. A study by Punzo D et al., 2016 reports that the downregulation of DDO genes promotes abnormal increases in free D-Asp levels in the brain, which causes neuroinflammation and cell death as age increases [41]. The increased D-Asp also causes dystrophic microglia, early caspase-3 activation, and cell death in cortical pyramidal neurons and dopaminergic neurons of the substantia nigra pars compacta [41]. Hence, the evidence and predisposition of lipufuscin granules in Ddo−/− knockdown brains confirm the significance of the DDO role in preventing neurodegenerative processes produced by non-physiological extracellular levels of free D-aspartate. Elevated levels of D-Asp act as agonists of the NMDA receptor, which is pathologically linked with PD [42]. Notably, MPTP showed an association between increased free D-aspartate and PD-like symptoms [43]. Currently, NMDA antagonists are suggested as an effective therapy for PD. But increased D-Asp has the tendency to activate NMDA [42]. However, the presence of DDO-catalyzed D-Asp reduces its level in the cellular environment. Thereby, maintaining the levels of DDO will help to maintain organelle connectivity as well as decrease the D-Asp that benefits PD treatment.

Thereby, we assessed the influence of anti-parkinsonism drugs on DDO through molecular docking. Of the 35 drugs analyzed, istradefylline had the highest affinity against DDO protein compared to other drugs. Alternatively, the lower affinity was noticed for amantadine with DDO, with no visible bond formation between the amantadine and DDO proteins. In general, istradefylline is used for patients with “off episodes”. This drug targets the adenosine A2A receptor as an antagonist and promotes dopaminergic activity by antagonizing adenosine in the basal ganglia [44,45,46]. Whereas, amantadine showed to decrease bradykinesia, rigidity, and tremor symptoms in PD individuals [45]. Amantadine shows a synergistic effect with levodopa, which promotes the conversion of dopamine with the help of striatal enzymes in the central nervous system [47,48,49]. Further, the MDS suggests that the DDO_istradefylline complex was highly stable based on the RMSD value when compared to the ligand-free and DDO_amantadine complexes [50]. Also, the high RMSD value of the ligand-protein complex suggests that the complex is unstable; ligand is not properly accommodated in the binding site of protein across the adopted MD simulation timeframes [51,52]. Upon simulation, the average RMSD of DDO-istradefylline is 0.18 when compared to DDO-amantadine (0.215) in docked structure. Similarly, RMSF values for DDO-istradefylline were high compared to other complexes; changes in the flexibility of the protein contributed significantly to the binding of the drug. Additionally, the number of hydrogen bonds formed within the complex was higher for DDO-istradefylline than for DDO-amantadine. More hydrogen bond formation relates to the high stability of the protein complex. However, no significant differences were observed for Rg and SASA between the complexes. Henceforth, the computational analysis suggests high protein stability was achieved due to the binding of istradefylline to DDO [53]. Further, to validate the off-target effect of anti-parkinsonism drugs, the PD participants under the istradefylline and amantadine treatments were recruited, and their DDO gene expression was assessed and compared with healthy controls. Notably, the participants under istradefylline with L-DOPA (group 1) therapy showed a significant decrease in expression of DDO when compared to individuals under amantadine (group 2) and the control group (group 3). Overall, our data clearly shows that istradefylline was able to manage their symptoms, but simultaneously, inhibition of DDO may negatively impact or disturb mitochondrial and ER connectivity, leading to the progression of neurodegeneration.

## 5. Conclusions

In conclusion, this study integrates a computational and molecular approach that identifies the D-aspartate oxidase (DDO), a crucial cytosolic protein essential for the communication between mitochondria and the endoplasmic reticulum for normal cellular function. Through molecular docking and MD simulation, our study also shows the off-target effects of anti-parkinsonism drugs that act upon the DDO protein. Among 35 drugs, istradefylline had high tendency to inhibit DDO protein, which leads to the loss of mitochondrial-ER connectivity. In contrast, amantidine had minimal effects on DDO, which could be a potential benefit in the treatment of PD. However, research in this direction is needed for further confirmation.

## Figures and Tables

**Table 1:** Clinical and demographic data of normal and PD participants under istradefylline or amantadine treatment.

Parameter	Istradefylline PD (*n* = 25)	Amantadine PD (*n* = 25)	Healthy Control (HC) (*n* = 25)	*p*-Value
Gender (Male/Female)	13/12	8/17	17/8	0.527
Age (mean ± SD)	56.51 ± 1.40	55.45 ± 1.81	51.18 ± 1.33	0.007
Age at onset (mean ± SD)	48.46 ± 10.23	46.72 ± 11.68		
Disease Duration Year (mean ± SD)	6.92 ± 3.42	6.89 ± 3.14		
UPDRS total (mean ± SD)	42.45 ± 19.02	41.35 ± 18.02		
UPDRS I	6.57 ± 4.15	6.47 ± 3.05		
UPDRS II	11.09 ± 8.36	11.29 ± 7.58		
UPDRS III	23.57 ± 14.34	23.71 ± 13.14		
UPDRS IV	1.85 ± 5.48	1.91 ± 5.18		
Hoehn and Yahr Scale	2.12 ± 0.60	2.32 ± 0.50		

**Table 2:** Primer Sequences and amplicon size.

Gene	Primer Sequence (5′->3′)		Ampliconic Size
DDO	Forward Sequence	GGAGCTGAAATCTCTCACCTGG	193
	Reverse Sequence	CTCATGGACACAGCACGGAT	
GAPDH	Forward Sequence	TGTCATCAACGGAAAGGC	183
	Reverse Sequence	GCATCAGCAGAAGGAGCA	

**Table 3:** Docking scores of anti-parkinsonism drugs against D-aspartate oxidase (DDO)cytosolic protein.

Sl. No	Drug ID	Drug Name	DOCKING SCORE (kcal/mol)
1	DB11757	Istradefylline	−9.073
2	DB06654	Safinamide	−8.862
3	DB00843	Donepezil	−8.861
4	DB00246	Ziprasidone	−8.381
5	DB06477	Sumanirole	−8.256
6	DB00494	Entacapone	−8.153
7	DB00490	Buspirone	−8.074
8	DB06454	Sarizotan	−7.918
9	DB01202	Lavetriacetam	−7.879
10	DB00323	Tolcapone	−7.703
11	DB00486	Nabilone	−7.455
12	DB00413	Pramipexole	−7.246
13	DB12551	Idazoxan	−7.082
14	DB06585	Fipamezol	−7.025
15	DB01367	Rasagiline	−6.788
16	DB00745	Modafinil	−6.766
17	DB00363	Clozapine	−6.711
18	DB05271	Rotigotine	−6.679
19	DB01235	Levodopa	−6.601
20	DB00571	Propranol	−6.551
21	DB06156	Tesofensine	−6.517
22	DB05814	Gpi-1485	−6.468
23	DB01224	Quetiapine	−6.333
24	DB00472	Fluoxetine	−6.31
25	DB00268	Ropinirole	−6.046
26	DB00190	Carbidopa	−6.025
27	DB00674	Galantamine	−6.012
28	DB01183	Naloxone	−5.886
29	DB04982	Talampanel	−5.768
30	DB00714	Apomorphine	−5.743
31	DB01043	Memantine	−5.605
32	DB00989	Rivastigmine	−5.486
33	DB00334	Olanzapine	−5.295
34	DB01037	Selegiline	−4.823
35	DB00915	Amantadine	−4.543

## Data Availability

Data are available on request.
